# Development of a single-cell derived MDSCs signature score for prognostic risk stratification and therapeutic decision guidance in breast cancer

**DOI:** 10.1016/j.tranon.2025.102605

**Published:** 2025-11-17

**Authors:** Jinbao Yin, Binbin Li, Hui Xiong, Jiepeng Gan, Lan Liang

**Affiliations:** a Department of Pathology, School of Basic Medical Sciences, Guangdong Medical University, Dongguan 523808, China; b Department of Biomedical Engineering, School of Biomedical Engineering, Guangdong Medical University, Dongguan 523808, China; c Department of Pathophysiology, School of Basic Medical Sciences, Guangdong Medical University, Dongguan 523808, China

**Keywords:** Breast cancer, MDSCs, Risk score model, TME, Immunosuppression

## Abstract

**Background:**

Myeloid-derived suppressor cells (MDSCs) function as critical immunosuppressive constituents within the breast cancer tumor microenvironment (TME). However, their molecular variability and clinical translation potential remain inadequately characterized.

**Methods:**

By combining single-cell RNA sequencing (scRNA-seq) with bulk multi-omics datasets, we screened and confirmed MDSCs signature genes specific to breast cancer. A prognostic risk scoring model was constructed using machine learning approaches and tested across multiple independent patient cohorts. The model’s predictive capacity for chemotherapy sensitivity, immunotherapy responsiveness, and TME features was systematically assessed.

**Results:**

After integrating the single-cell datasets GSE161529 and GSE176078, we identified 12,767 MDSCs along with their 209 characteristic genes. To ensure the reliability of our results, we employed various analytical methods and utilized diverse data for validation. From this signature, a 5-gene risk score model comprising BCL2A1, GDI2, GRINA, RNASE1, and SERPINA1 was constructed, demonstrating robust prognostic stratification with a 1- to 10-year overall survival (OS) AUC exceeding 0.6 and independent predictive value. High-risk patients exhibited characteristic features of an immunosuppressive tumor microenvironment (TME), including increased M2 macrophages and regulatory T cells, alongside diminished cytotoxic T lymphocyte activity. These patients also showed poor responses to both chemotherapy and immunotherapy.

**Conclusion:**

This investigation marks the initial systematic characterization of a new MDSCs gene signature in breast cancer, alongside the establishment of an MDSCs-associated marker scoring framework with multi-aspect clinical translation capability, thereby linking MDSCs fundamental biology to precision oncology in this cancer type.

## Highlights

• Novel MDSCs Gene Signature: First systematic identification of 209 breast cancer-specific MDSCs signature genes via integrated single-cell RNA-seq and bulk multi-omics analysis, providing a molecular basis for MDSCs characterization in the tumor microenvironment (TME).
• Robust Prognostic Model: Development of a 5-gene (BCL2A1, GDI2, GRINA, RNASE1, SERPINA1) risk score model with strong prognostic value, achieving 1–10 year overall survival AUC > 0.6 and independent predictive capacity across TCGA-BRCA, METABRIC, and SCANB cohorts.
• TME and Therapy Response Stratification: High-risk patients exhibit an immunosuppressive TME (enriched M2 macrophages/regulatory T cells, reduced cytotoxic T lymphocyte activity) and poor responses to chemotherapy/immunotherapy, while low-risk patients show heightened drug sensitivity and favorable immunotherapeutic outcomes.
• Clinically Translatable Tools: Construction of a nomogram integrating the MDSCs risk score with clinicopathological factors (age, TNM stage) to enhance personalized survival prediction, validated by calibration plots and decision curve analysis.
• Precision Oncology Implications: The risk score system enables tri-dimensional clinical guidance—prognostic stratification, chemotherapy sensitivity assessment, and immunotherapy response prediction—supporting MDSCs-targeted therapeutic strategies in breast cancer.

## Introduction

Breast cancer, the most common malignant neoplasm impacting global female health, contributes to more than 2.3 million annual newly diagnosed instances and ranks as the primary driver of cancer-associated mortality in females [1]. The pathophysiology of breast cancer is intricate, driven by a multidimensional interaction among genetic susceptibilities, dysregulated epigenetic processes, and disequilibrium in the tumor microenvironment. Notwithstanding notable progress in precision medicine that has boosted the 5-year survival probability of patients with early-stage breast cancer to over 90%, neoplastic heterogeneity and therapy resistance remain major obstacles, with around 40% of early-stage cases developing into advanced disease [2,3]. Notably, the five-year survival rate for patients with metastatic breast cancer remains below 30% [4]. This urgent dilemma underscores the necessity to investigate resistance mechanisms, particularly from the perspective of the tumor microenvironment, and to develop innovative prognostic assessment systems.

The tumor microenvironment operates as a dynamically developing ecological system, where MDSCs exert a critical dual regulatory function in facilitating neoplastic progression [5]. MDSCs drive tumorigenesis by releasing immunosuppressive mediators such as nitric oxide and IL-10, which impair T and NK cell activity. Furthermore, they promote metastasis via VEGF-dependent angiogenesis and epithelial-mesenchymal transition (EMT). Notably, MDSCs infiltration levels exhibit significant correlations with chemoresistance and reduced responsiveness to immunotherapy, underscoring their potential as key targets for prognostic assessment [6]. However, current marker systems (e.g., CD11b/Gr-1) are constrained by variability within the tumor microenvironment, hampering precise characterization of MDSCs functional diversity. Though advances in single-cell sequencing have driven the discovery of novel MDSCs markers (such as IL1B/CCR1 in gastric carcinoma and CD84 in breast carcinoma), absence of a holistic gene expression profile persistently impedes the formulation of targeted therapeutic strategies [7,8].

This research intends to combine single-cell RNA-seq and bulk RNA-seq datasets to develop a distinctive gene signature for MDSCs in breast cancer, thereby building a prognostic model. Through multi-omics validation, this model will enable effective assessment of patient prognostic stratification, chemosensitivity, and immunotherapeutic response, elucidating the molecular characteristics that help differentiate risk subgroups. The findings from this research will provide a new set of biomarker candidates for precision treatment of breast cancer and establish a foundation for translational research targeting MDSCs.

## Materials and methods

### Data acquisition and organization

**Fig. 1:**
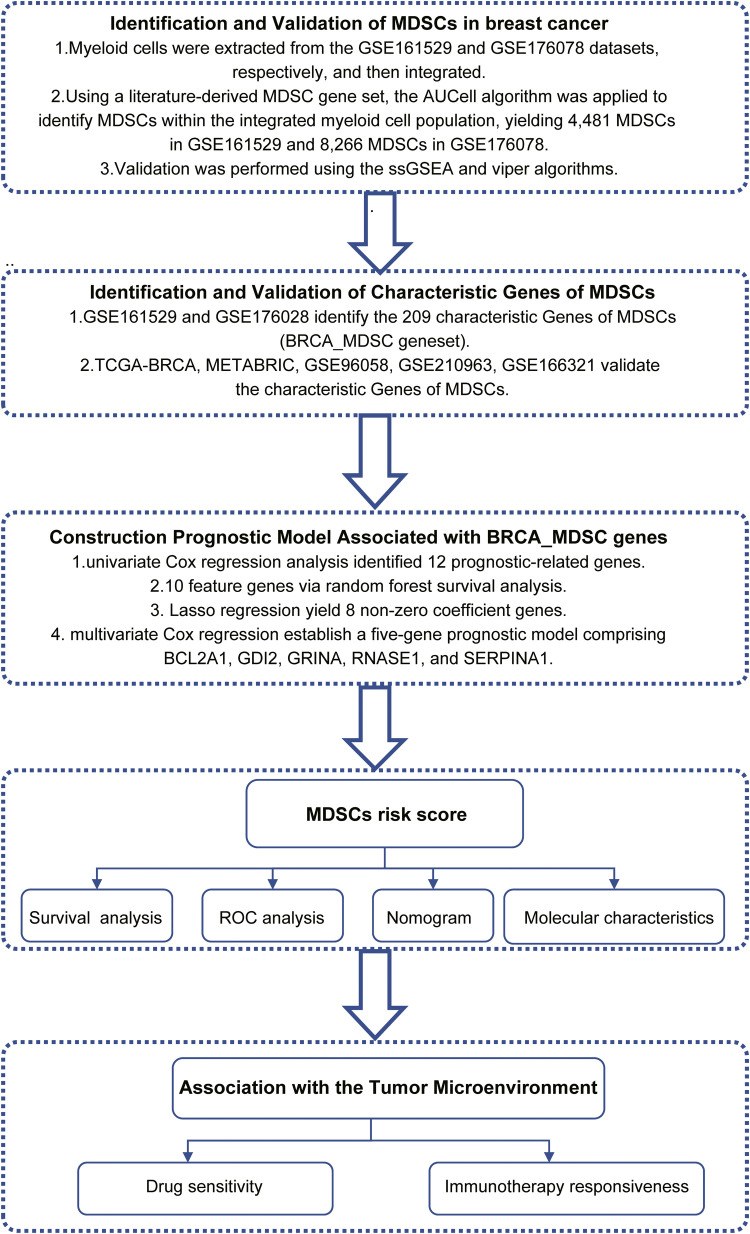
Methodological workflow of the present study.

Our investigation incorporates multi-source omics datasets, including RNA-seq data (TPM) from the TCGA BRCA cohort. A total of 1,118 tumor specimens in this cohort were obtained via the "TCGAbiolinks" package, among which 1,091 samples with complete prognostic and clinical characteristics were utilized in the present study [9]. Moreover, expression datasets and clinical records derived from 32 studies in the GEO repository were retrieved, downloaded, and collated using the "GEOquery" package [10]. Microarray data from the METABRIC cohort, consisting of 1,980 samples, were sourced from the cBioPortal website. To address batch effects across different platforms, we employed the ComBat algorithm for correction [11]. Single-cell transcriptomic data were acquired from the NCBI GEO, while characteristic gene sets were defined based on existing literature. Comprehensive dataset specifications are documented within the Supplementary Table1, with a schematic summary of the analytical workflow utilized in this investigation illustrated in Fig. 1.

### Single-cell dataset curation and analysis

We selected the single-cell transcriptomic datasets GSE176078, GSE169246, and GSE210963, all of which feature comprehensive cell type annotations. For the GSE161529 and GSE166321 dataset, we conducted a re-analysis utilizing the “Seurat” R package, following a multi-step workflow [12]: (1) Rigorous quality control procedures were applied to filter out suboptimal cells, which were characterized by ≤200 detected genes, <1,000 unique molecular identifier (UMI) counts, or mitochondrial gene content surpassing 20%; (2) LogNormalize normalization was implemented, followed by the extraction of 3,000 highly variable genes (HVGs); (3) leveraging the top 30 principal components (PCs), a K-nearest neighbor (KNN) graph (k=20) was built to enable unsupervised clustering via the Louvain algorithm, with cell subtype annotation subsequently conducted using established marker genes.

To enable cross-study harmonization of single-cell data sets, canonical correlation analysis (CCA) was applied to identify mutual nearest neighbors (anchors), maintaining 30 integration dimensions to reduce technical batch effects [13]. Gene set enrichment levels were quantified using the AUCell algorithm, where activity scores were represented as area under the curve (AUC) values [14]. To rectify technical noise induced by gene dropout events, nonlinear imputation was implemented on the expression matrix of highly variable genes via the MAGIC algorithm [15]. Additionally, gene regulatory networks were reconstructed using the ARACNe-AP algorithm, and transcriptional regulator activity was inferred through the VIPER framework [16].

### Prognostic signature construction and validation

Genes associated with prognosis were determined via univariate Cox regression analysis, with a significance cutoff set at *P* < 0.05. The top 10 feature genes were further selected using a random forest model based on importance scores. Subsequently, a LASSO regression algorithm was applied to screen genes with non-zero coefficients, thereby constructing a multivariate Cox regression-derived prognostic risk score model defined by the following equation: prognostic risk score = Σ (gene expression value × corresponding regression coefficient).

Patients were divided into high-risk and low-risk subgroups, with the median risk score serving as the cutoff value. The predictive capability of the model was assessed via time-varying receiver operating characteristic (ROC) analysis curves, computing the AUC (area under the curve) for 1-, 3-, 5-, 8-, and 10-year survival predictions. Survival differences between subgroups were validated using Kaplan-Meier survival analysis and log-rank tests [17].

### Nomogram construction

Clinicopathological characteristics and risk score showing significant correlation with the OS (with *P* < 0.05 as the significance criterion) were detected via univariate Cox regression modeling. Independent prognostic indicators were ascertained through multivariate Cox regression analysis, enabling the construction of an integrated nomogram model via the "rms" R package [18]. Nomogram predictive capacity was analyzed using calibration plots for evaluating predictive precision, time-dependent decision curve analysis (DCA) to define clinical net benefit thresholds, and time-dependent ROC plots to measure AUC metrics at specified time intervals [19].

### Enrichment analysis

Analyses of functional enrichment, encompassing KEGG pathway and Gene Ontology (GO) term assessments, were performed via the "clusterProfiler" R package, with statistical significance defined as an adjusted p-value (q-value) < 0.05 [20]. Within gene set enrichment analysis (GSEA), terms exhibiting significant enrichment were determined using the absolute normalized enrichment score (|NES| > 1) combined with a nominal *P*-value < 0.05 [21]. Gene set variation analysis (GSVA) was applied to systematically assess pathway activity variations among experimental groups, elucidating the functional relevance of recognized gene signatures [22].

### Tumor microenvironment profiling and immunotherapy response prediction

Key elements within the tumor microenvironment (TME) were quantitatively evaluated via the ESTIMATE algorithm [23]. Immune infiltration was characterized by systematically quantifying the proportions of 22 immune cell subtypes through the CIBERSORT deconvolution algorithm [24].

To predict immunotherapy responses, critical parameters including the CD8+/Treg ratio and the quantity of activated cytotoxic T lymphocytes (CTLs) were computed via the "IOBR" R package, leveraging quantified immune cell infiltration characteristics [25]. These metrics were combined with the Tumor Immune Dysfunction and Exclusion (TIDE) algorithm to evaluate features of immune evasion, offering insights into the probability of response to immunotherapy [26].

### Transcriptomic molecular subtyping and subgroup heterogeneity analysis

Molecular subtyping was conducted using the ConsensusClusterPlus algorithm, leveraging multi-gene set expression profiles [27]. Parameters included 1,000 bootstrap resampling iterations, pItem = 0.8 (sample sampling proportion), pFeature = 1 (full feature inclusion), and iteratively testing the number of clusters (k) from 2 to 10. The optimal cluster number was determined through a comprehensive approach that integrated the elbow method with silhouette coefficient analysis. Cluster visualization was achieved using the t-SNE package, facilitating the effective representation of complex data structures. This analysis identified stable molecular subtypes, enabling systematic characterization of inter-subgroup heterogeneity in molecular expression features. Diagnostic receiver operating characteristic (ROC) curves were generated via the “pROC” package to assess diagnostic efficacy for binary clinical endpoints, where AUC values reflect discriminative ability [28].

### Molecular subgroup chemotherapy response prediction

To assess therapy response variability, drug sensitivity datasets derived from the Genomics of Drug Sensitivity in Cancer (GDSC) repository were incorporated, and predictive models were constructed using the "pRophetic" package [29]. These models quantitatively evaluated patient-specific sensitivity parameters, including IC50 (half-maximal inhibitory concentration) values for diverse therapy drugs.

### Statistical analysis

All statistical computations were executed through the combined application of the R programming language and RStudio integrated development environment. Pairwise group comparisons were conducted via the Wilcoxon rank-sum test, whereas multigroup differences utilized one-way analysis of variance (ANOVA). Associations between variables were evaluated through Pearson correlation analysis, executed with the cor.test function. Statistical significance was set at a two-sided *P*-value < 0.05, and results are represented by asterisks as follows: *p < 0.05, **p < 0.01, and ***p < 0.001.

## Results

### Comprehensive characterization and prognostic modeling of myeloid-derived suppressor cells in breast cancer

**Fig. 2:**
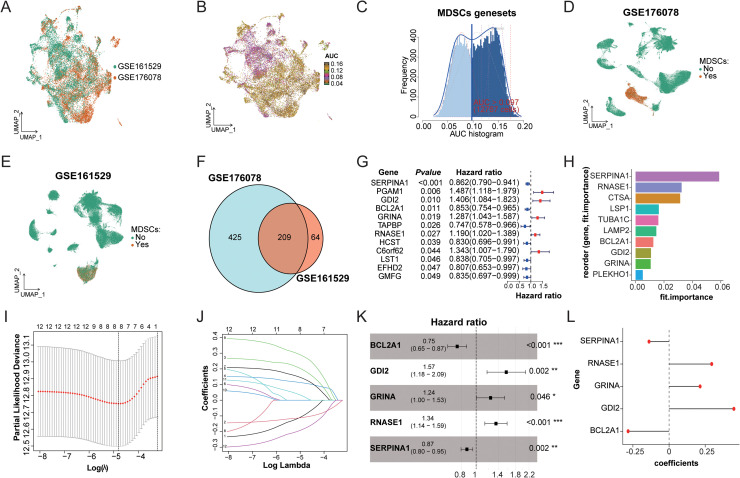
**Identification of signature genes for breast cancer MDSCs and development of a prognostic risk scoring model.** (A) UMAP clustering map of 21,651 myeloid cells Integrated from GSE161529 and GSE176078 datasets. (B-C) Spatial distribution of AUC scores based on literature-reported MDSCs signature gene sets within the integrated myeloid cell map (B) and frequency distribution of AUC values. (D-E) UMAP distributions of MDSCs subpopulations identified by the MDSCs signature gene set in GSE176078 (D) and GSE161529 (E) datasets. (F) Venn diagram analysis of MDSCs signature genes across GSE161529 and GSE176078 datasets. (G) Univariate Cox regression modeling for identifying prognosis-associated genes within the BRCA-MDSCs gene set. (H) Variable importance ranking of the 12 prognosis-related genes using the random forest algorithm. (I-J) Lasso regression analysis for further screening of non-zero coefficient genes. (K) Forest plot demonstrates multivariate Cox regression analysis based on LASSO results. (L) Distribution of hazard coefficients for the 5 core genes (BCL2A1, GDI2, GRINA, RNASE1, and SERPINA1) in the prognostic model.

This study integrated single-cell datasets GSE161529 (11,976 myeloid cells) and GSE176078 (9,675 myeloid cells) to construct a comprehensive atlas of 21,651 myeloid cells using canonical correlation analysis (CCA) in Seurat package (Fig. 2A). Based on a curated set of 312 MDSCs signature genes compiled from previously reported literature (Supplementary Table 2), this study employed the AUCell algorithm for the identification and quantitative analysis of MDSCs in breast cancer samples. Through optimized parameter settings (threshold = 0.097), a total of 12,767 MDSCs were successfully identified, including 4,481 derived from the GSE161529 dataset and 8,286 from the GSE176078 dataset (Figs. 2B-C, S1A).

Validation analyses indicated that the single-sample gene set enrichment analysis (ssGSEA)-derived scores of MDSCs showed significantly elevated levels compared with those of other myeloid subpopulations (no MDSCs, *P* < 0.001, Fig. S1B), with notable upregulation of protein activity for CD14, CD33, IGTAM, S100A8 and S100A9 in MDSCs, as determined by the viper algorithm (*P* < 0.01, Fig. S1C).

The characteristic genes were filtered based on criteria of log2 fold-change (log2FC) > 0.3, percentage of cells expressing the gene in group 1 (pct.1) > 0.4, and pct.2 < 0.4, resulting in 224 characteristic genes from GSE161529 and 634 from GSE176078, with 209 genes common to both datasets, which were subsequently designated as the BRCA-MDSCs gene set (Fig. 2D-F, Supplementary Table 3). Bulk multi-cohort validation revealed a significant positive correlation between this gene set and established MDSCs characteristics comprising 312 genes (r = 0.82, *P* = 2.22e-16) (Fig. S1D).

Within drug validation assays, the GSE210963 dataset revealed a notable decrease in AUCell scores among the ibrutinib-treated cohort (*P* < 2.22e-16, Fig. S1E-F). Similarly, the GSE166321 dataset confirmed a significant downregulation of BRCA-MDSCs characteristic genes in the entinostat treatment group (normalized enrichment score, NES = -1.45, FDR q = 0.000, Fig. S1G), accompanied by a significant decrease in AUCell scores at the single-cell level (*P* < 2.22e-16, Fig. S1H-I). These findings suggest that the 209 genes effectively represent MDSCs features and provide a molecular basis for targeted therapy in breast cancer.

Based on the TCGA-BRCA cohort, this study developed a prognostic model related to MDSCs through a multi-stage selection process. Preliminary univariate Cox proportional hazards regression analysis pinpointed 12 prognosis-associated genes within the 209 MDSCs feature gene pool (*P* < 0.05, Fig. 2G). This subset was subsequently narrowed to 10 feature genes via random forest survival analysis (Fig. 2H). Lasso regression (λ = 0.0082) refined the selection further, yielding 8 non-zero coefficient genes, ultimately leading to the establishment of a five-gene prognostic model comprising BCL2A1, GDI2, GRINA, RNASE1, and SERPINA1 through multivariate Cox regression (Fig. 2I-K). The risk index is determined by the subsequent equation: Risk Score = Σ (Gene Expression × Coefficient). In this equation, GDI2 (coefficient = 0.448813946), RNASE1 (coefficient = 0.295240562), and GRINA (coefficient = 0.214979736) are identified as positive contributors, whereas SERPINA1 (coefficient = -0.138540766) and BCL2A1 (coefficient = -0.283156346) serve as negative contributors (Fig. 2L).

### Prognostic significance of MDSCs risk scoring and nomogram in breast cancer management

**Fig. 3:**
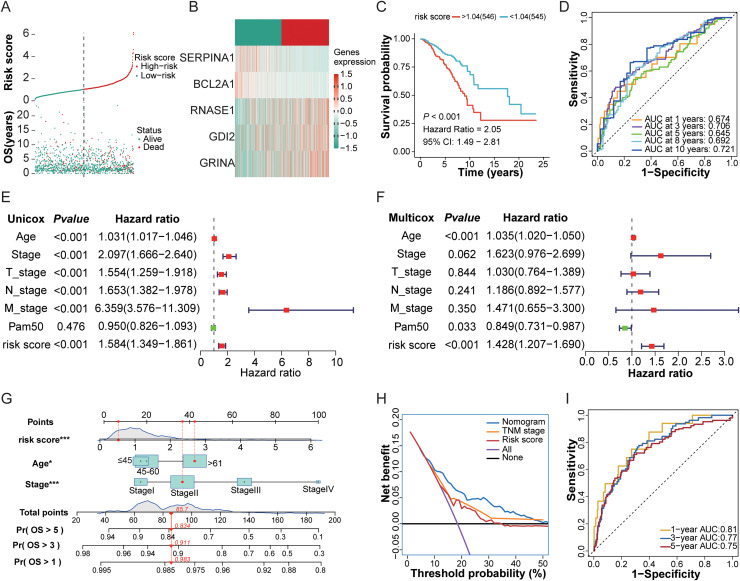
**Prognostic Assessment and Model Verification of the MDSCs Risk Score within the TCGA_BRCA Cohort**. (A) Samples were assigned to high-risk or low-risk subgroups according to the median risk score threshold. (upper panel), with a scatter plot illustrating survival outcomes across these groups (lower panel). (B) Differential expression patterns of the 5 core genes in high-risk versus low-risk patient cohorts. (C) Kaplan-Meier survival curves were plotted for the high-risk and low-risk groups, with log-rank test applied. (D) Time-varying ROC curve analysis evaluated the predictive performance of the risk score for overall survival across distinct time points. (E-F) Both univariate and multivariate Cox regression models validated the risk score's prognostic independence. (G) A prognostic nomogram was constructed to enable personalized survival prognostication, integrating tumor stage, age, and the MDSCs risk score. (H) Decision curve analysis validated the clinical applicability of the nomogram. (I) Time-dependent ROC analysis validated its predictive efficacy for OS using the nomogram.

As depicted in Fig. 3A-B, within patients classified into the high-risk subgroup using the median risk score as the cutoff, the expression of GDI2, GRINA, and RNASE1 displayed significant upregulation, whereas BCL2A1 and SERPINA1 presented a downregulated expression pattern. Kaplan-Meier survival curve analysis revealed that subjects categorized as high-risk displayed a notably abbreviated overall survival duration (*P* < 0.001), accompanied by a 71.2% elevation in mortality rate relative to the low-risk group (17.8% vs. 10.4%, Fig. 3C). This risk scoring system demonstrated robust prognostic predictive ability across two independent validation cohorts: METABRIC (HR = 1.33, 95% CI 1.18-1.49), and SCANB (HR = 1.44, 95% CI 1.16-1.78), all achieving statistical significance (*P* < 0.001, Fig. S2A-B). Notably, this model also displayed significant predictive value for recurrence-free survival (RFS; HR = 1.21, *P* < 0.001) and distant metastasis-free survival (DMFS; HR = 1.54, *P* < 0.001) (Fig. S2C-D). Time-varying receiver operating characteristic (ROC) assessment further validated its consistent predictive performance, showing area under the curve (AUC) values spanning 0.674 to 0.721 across a 1- to 10-year timeframe (Fig. 3D).

Multi-cohort, univariate, and multivariate Cox proportional hazards regression models validated the independent prognostic significance of the MDSCs risk score. Within the TCGA-BRCA dataset, both univariate and multivariate Cox regression models demonstrated that the risk score and age served as independent prognostic indicators (both *P* < 0.05, Fig. 3E-F). This observation was further confirmed across the METABRIC and SCANB datasets (Fig. S2E-H).

Subsequently, we constructed a prognostic nomogram integrating TNM stage, age, alongside the MDSCs risk score (Fig. 3G). Calibration plot analysis revealed strong consistency between predicted survival probabilities and empirically observed survival events, while decision curve analysis confirmed the model's clinical applicability (Figs. S2I, 3H). Cross-cohort time-dependent ROC evaluations indicated that within the TCGA-BRCA dataset, the nomogram achieved AUC values of 0.81 (1-year OS), 0.77 (3-year OS), and 0.75 (5-year OS) for overall survival prediction, corresponding AUCs in METABRIC were 0.76 (1-year), 0.63 (3-year), and 0.65 (5-year), and in SCANB, these values reached 0.71 (1-year), 0.65 (3-year), and 0.65 (5-year) (Figs. 3I, S2J-K).

### Tumor microenvironment characterization in MDSCs risk stratification

**Fig. 4:**
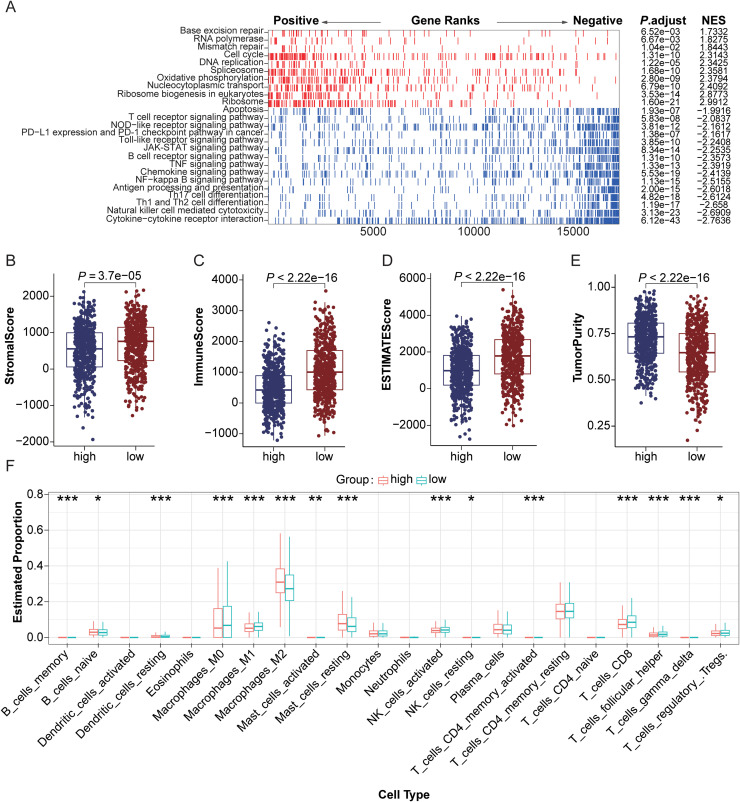
Subgroup molecular characteristics of the MDSCs risk score and its correlation with the tumor immune microenvironment. (A) Relational analysis of MDSCs risk score with KEGG pathways within the SCANB cohort. (B-E) Comparison of stromal score (B), immune score (C), ESTIMATE score (D), and tumor purity (E) across high- vs. low-risk subgroups in the TCGA-BRCA cohort (Wilcoxon rank-sum test). (F) Relative distribution pattern of 22 tumor-infiltrating immune cell types across high-risk versus low-risk score subpopulations within the SCANB study cohort.

Based on differential expression analysis of the TCGA-BRCA cohort, we identified 292 core genes that effectively distinguish between high and low MDSCs risk score subgroups (Supplementary Table 4). Functional annotation enrichment analysis identified notable correlations for these genes with biological processes and signaling pathways involved in adaptive immune regulation, particularly in the activation of B and T lymphocytes as well as NK cell-dependent cytotoxicity (Fig. S3A-B). Verification via gene set enrichment analysis within the SCANB cohort further confirmed these observations, showing a marked inverse association between the MDSCs risk score and anti-tumor immunity-related signaling pathways, including Toll-like receptor and NOD-like receptor signaling (Fig. 4A).

Moreover, evaluation of tumor microenvironment traits via the ESTIMATE algorithm demonstrated that specimens with elevated MDSCs risk scores showed notably diminished stromal, immune, and total scores, which suggests increased tumor purity and decreased infiltration of stromal and immune cells (Fig. 4B-E, *P* < 0.001). CIBERSORT-based immune cell infiltration assessment further demonstrated marked accumulation of immunosuppressive cell populations, including M0/M2 macrophages, follicular helper T lymphocytes, and regulatory T cells (Tregs), within the high-risk MDSCs subgroup (Fig. 4F, *P* < 0.05).

Moreover, comparative analysis of the cellchat interaction network based on the GSE176078 dataset revealed that MDSCs in the high MDSCs risk score subgroup significantly enhance pro-tumor interactions with T/B lymphocytes, cancer-associated fibroblasts, and endothelial cells. This enhancement is mediated through the activation of signaling axes such as TGF-β, VEGFA, and LGALS9-HAVCR2, thereby facilitating immune evasion and promoting tumor angiogenesis (Fig. S4A-D).

### MDSCs risk score efficiently directs prognostication of response to chemotherapy

**Fig. 5:**
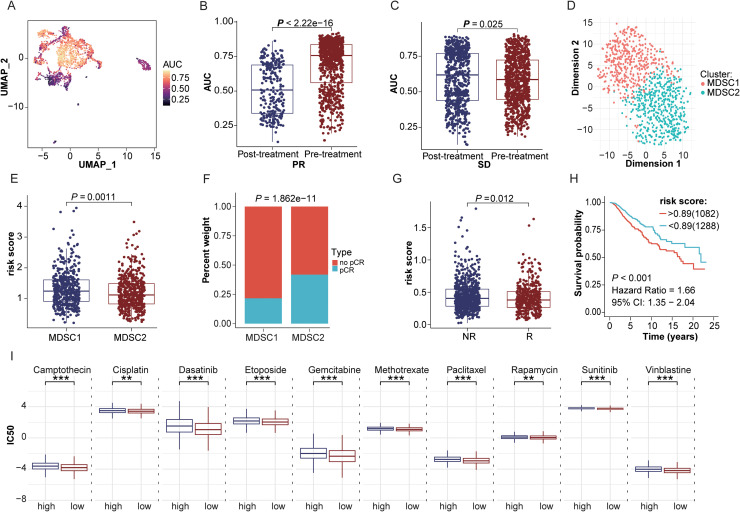
Exploratory examination of the association between MDSCs risk scoring and patient responsiveness toward pharmacological intervention. (A) Spatial distribution characteristics of AUCell activity scores of the BRCA-MDSCs gene set in the GSE169246 treatment cohort. (B-C) Paired assessments of AUCell activity scores for the BRCA-MDSCs gene set across pre- versus post-treatment intervals within the PR subgroup (B) and SD subgroup (C). (D) Consensus clustering outcomes for the GSE194040 cohort using the BRCA-MDSCs gene set, depicting the distribution of cluster members and the number of samples in each subgroup. (E) Comparison of MDSCs risk score differences between different clustering clusters in the GSE194040 cohort. (F) Correlation analysis between different clustering clusters and treatment response in the GSE194040 cohort. (G) Examination of MDSCs risk scoring value disparities across treatment-responsive vs non-responsive cohorts following multi-dataset integration from GEO treatment repositories. (H) Kaplan-Meier Survival analysis of MDSCs risk scores in patients receiving chemotherapy across the TCGA-BRCA, METABRIC, and SCANB cohorts (log-rank test). (I) Exploratory assessment of the association between high- vs low-risk stratification clusters with chemotherapeutic agent responsiveness within the TCGA-BRCA dataset.

Drawing from AUCell scoring analysis of BRCA-MDSCs gene set, our findings revealed that myeloid cell scores among GSE169246 cohort individuals achieving complete response (CR) exhibited a notably reduced level compared to those presenting with stable disease (SD) (*P* < 0.05), suggesting an association between MDSCs infiltration and chemotherapy resistance (Fig. 5A–C). Using unsupervised clustering analysis of the BRCA-MDSCs gene set, 988 chemotherapy samples from the GSE194040 cohort were categorized into high (Cluster 1, n = 467) and low (Cluster 2, n = 521) MDSCs risk score subgroups. Notably, low MDSCs risk score subgroup displayed a markedly elevated treatment response relative to the high-risk cluster (*P* = 1.862e-11, Fig. 5D–F). Verification via data integration across seven GEO databases (n = 1,123) demonstrated that MDSCs risk scores among treatment-responsive individuals exhibited a notably reduced level compared to those within the non-responsive cohort (*P* = 0.012, Fig. 5G). Additionally, Kaplan-Meier survival analysis incorporating data from TCGA-BRCA, METABRIC, and SCANB cohorts with available chemotherapy information revealed that individuals with elevated MDSCs risk scores exhibited worse prognostic outcomes when compared to counterparts with reduced MDSCs risk scores (HR =1.66, 95% CI: 1.35-2.04, Fig. 5H). Additionally, drug sensitivity predictions indicated that the low MDSCs score group exhibited significantly reduced IC50 values for various chemotherapeutic and targeted agents, including Camptothecin, Cisplatin, Dasatinib, Etoposide, Gemcitabine, Methotrexate, Paclitaxel, Rapamycin, unitinib and Vinblastine (Fig. 5I).

### Exploratory evaluation of the relationship between MDSCs risk score and immunotherapy response capability

**Fig. 6:**
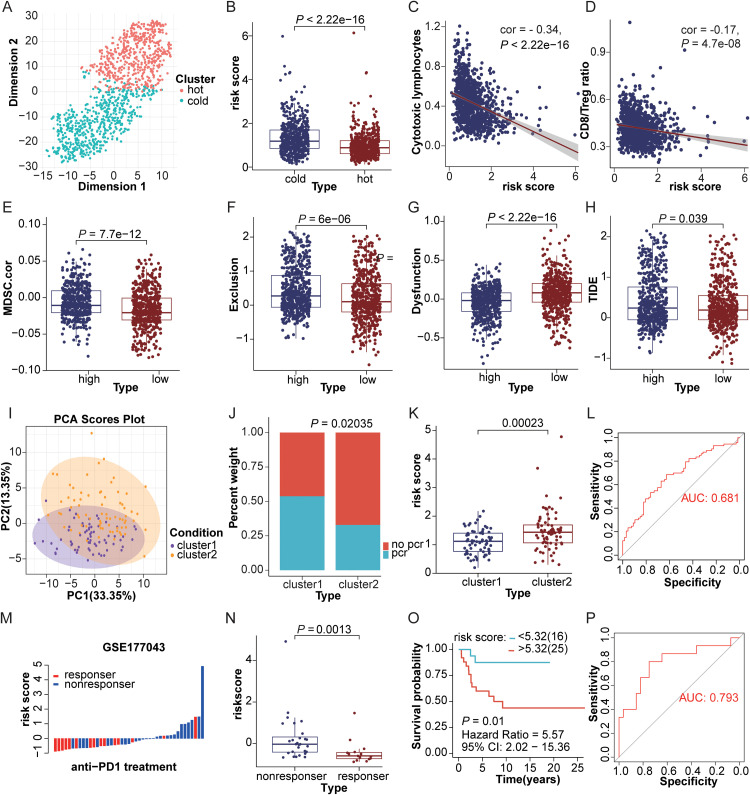
Association assessment of MDSCs risk score with immunotherapeutic responsiveness in breast cancer cohorts. (A) Sample distribution patterns and count metrics for cold vs. hot tumor subpopulations derived from consensus-driven clustering profiling of the TCGA-BRCA dataset. (B) MDSCs risk score disparities across cold vs. hot tumor subpopulations within the TCGA-BRCA dataset. (C-D) Correlative evaluations of MDSCs risk scores with cytotoxic T lymphocyte (CTL) functional activity (C) along with CD8⁺ T cell-to-Treg cell proportionality (D) within the TCGA-BRCA dataset. (E-H) Cross-group comparisons of MDSC correlation (E), Exclusion index(F), T cell dysfunction metric (G), and TIDE evaluation index (H) were performed between high versus low MDSCs risk categories within the TCGA-BRCA patient cohort. (I). Principal component analysis (PCA) visualization depicts the consensus clustering pattern derived from 79 immune checkpoint-associated genes across the GSE173839 alongside GSE194040 datasets. (J) Response rates to immunotherapy were compared between immune checkpoint clusters 1 and 2. (K) Differences in MDSCs risk scores between immune checkpoint clusters 1 and 2. (L) The diagnostic efficacy of the MDSCs risk score in distinguishing between immune checkpoint clusters 1 and 2. (M) Waterfall plot depicts the distribution pattern of MDSCs risk score values and ICI response status within the GSE177043 immunotherapy patient cohort. (N) Variations in MDSCs risk score values across responder versus non-responder subgroups within the GSE177043 patient cohort. (O) Kaplan-Meier survival curve comparison of high versus low MDSCs risk categories following immunotherapy in the GSE177043 dataset(P). Receiver Operating Characteristic (ROC) curve analysis was utilized to examine the predictive utility of the MDSCs risk score in forecasting responsiveness to immunotherapy among breast cancer cases within the GSE177043 dataset.

Utilizing genes associated with the hot tumor phenotype (Supplementary Table 5), we performed unsupervised clustering to categorize the TCGA-BRCA cohort into hot and cold tumor subtypes. MDSCs risk scores within the hot tumor subgroup showed a significantly lower magnitude compared to those in the cold tumor subgroup (*P* < 0.001, Fig. 6A, B), indicating a potential link to immunotherapy responsiveness. Subsequent analyses demonstrated that MDSCs risk scores exhibited significant negative correlations with cytotoxic T lymphocyte activity levels (r = -0.34, *P* < 0.001) and CD8+ T cell to Treg cell ratios (r = -0.17, *P* < 0.001) (Fig. 6C, D), suggesting that reduced scores might potentiate antitumor immune reactivity.

The TIDE scoring system was employed to assess the immune suppressive microenvironment, revealing that the low MDSCs risk score subgroup exhibited higher T cell dysfunction scores, alongside lower correlations with MDSCs, tumor immune rejection scores, and TIDE scores, this indicates a weaker immune suppressive environment in this subgroup (Fig. 6E–H). To validate clinical relevance, we integrated the GSE173839 and GSE194040 datasets, which included information on anti-PD-1/PD-L1 therapies and chemotherapy, and conducted clustering analysis of 79 immune checkpoint genes (Supplementary Table 5). This analysis identified two subtypes: The immune checkpoint cluster1(encompassing 67 samples) versus the immune checkpoint cluster2 containing 73 samples) (Fig. 6I). Exploratory cumulative distribution evaluation demonstrated that individuals within immune checkpoint cluster1 had a significantly higher treatment response rate compared to those in immune checkpoint cluster2 (*P* = 0.02035), with notably lower MDSCs risk scores (*P* = 0.00023) (Fig. 6J, K). Receiver operating characteristic (ROC) curve-based validation established the MDSCs prognostic risk score's robust capacity to distinguish between the two subtypes (AUC = 0.681, Fig. 6L). Within the GSE177043 patient cohort, which was exclusively composed of individuals undergoing anti-PD-1/PD-L1 immunotherapy, a notable decline in the count of subjects attaining therapeutic responses was detected as MDSCs risk score increased (Fig. 6M). Responders demonstrated significantly lower risk score compared to non-responders (*P* = 0.0013, Fig. 6N). Extended Kaplan-Meier survival curve analysis revealed a notable disparity in overall survival outcomes across high- versus low-MDSCs score subpopulations, where individuals categorized into the high-MDSCs risk stratum displayed inferior prognostic trajectories when contrasted with the low-MDSCs score counterpart group (HR = 5.57, *P* = 0.01, Fig. 6O). Follow-up ROC curve analysis additionally confirmed that MDSCs scores function as a robust predictive indicator for treatment responsiveness in breast cancer immunotherapy (AUC = 0.793, Fig. 6P).

## Discussion

Within the investigation of breast cancer's intricate nature, the dynamic progression and marked variability of myeloid-derived suppressor cells (MDSCs) within the tumor microenvironment (TME) emerge as especially pivotal. While a substantial body of research has validated the critical function of MDSCs in breast cancer initiation, progression, and therapeutic modulation, there persists a pronounced deficit in the characterization of MDSCs-specific gene signatures and their multifaceted application potential in patient prognostic evaluation and treatment decision-making. To bridge this gap, this study integrates multi-omics data and employs various bioinformatics analysis methods to systematically identify, for the first time, a characteristic gene set of MDSCs in the breast cancer TME and further constructs a corresponding risk scoring system. This advancement not only provides new tools for elucidating the immune-suppressive TME mediated by MDSCs but also offers critical insights into their comprehensive biological characteristics and clinical translational potential.

Through the combination of single-cell sequencing and bulk sequencing datasets, this study systematically screened a characteristic gene set of MDSCs comprising 209 genes, overcoming limitations in the understanding of MDSCs molecular characteristics as identified in previous studies. This gene set reveals a multi-level functional regulatory network from molecular mechanisms to functional phenotypes. At the molecular level, transcription factors such as STAT1, CEBPB, HIF1A, and REL, collectively drive MDSCs expansion, while chemokines like CCL3, CCL4, CCL8 and IL1B regulate their migration within the tumor microenvironment [[30], [31], [32], [33], [34], [35]]. At the functional phenotypic level, immune checkpoint molecules, metabolic reprogramming and lysosomal enzymes collectively mediate the enhancement of the immune suppressive activity of MDSCs [[36], [37], [38], [39]]. Furthermore, FGL2 also promote tumor immune evasion by directly blocking T-cell activation [40,41]. Notably, the high expression of phagocytic receptors such as the FCGR family and MSR1 suggests that MDSCs may interact functionally with other cells through phagocytosis [42,43]. Despite persisting uncertainty surrounding the definition, origin, and functional properties of MDSCs, analyzing their characteristic molecular modules not only yields molecular markers for the accurate identification of MDSCs within the breast cancer microenvironment but additionally establishes theoretical foundations to underpin targeted therapeutic modalities focused on metabolic reprogramming, epigenetic regulation, and immune checkpoint blockade.

Leveraging the identified MDSCs characteristic gene signatures, this investigation applied machine learning methodologies to construct a prognostic model comprising BCL2A1, GDI2, RNASE1, SERPINA1, and GRINA. The selection of this gene set reflects its dynamic regulatory network across different molecular functional dimensions: BCL2A1, acting as a key apoptosis-inhibitory component among Bcl-2 family members, is commonly characterized as a distinct oncogene and demonstrates a robust correlation with drug tolerance to chemotherapeutic agents as well as targeted therapeutics [44]. GDI2, functioning as a component within the GDP dissociation inhibitor (GDI) family, exerts a pivotal function in sustaining cellular homeostasis by modulating Rab GTPases implicated in vesicle trafficking. Meanwhile, its dual functions in immunoregulation and cancer have also garnered extensive attention [45]. RNASE1, a member of the ribonuclease A superfamily, exhibits bidirectional regulatory properties in the tumor microenvironments of various cancer types, which may be associated with its RNA degradation activity and the heterogeneity of the tumor microenvironment [46]. GRINA is characterized as a target gene regulated by the unfolded protein response (UPR) pathway, and it mediates cellular apoptosis through modulation of endoplasmic reticulum calcium homeostasis [47]. SERPINA1, functioning as a serine protease inhibitor, is known to exhibit robust links to tumor invasion, metastasis, as well as proliferation [48]. Nevertheless, this gene was identified as a protective factor in the current study, suggesting its functional role in tumors may be more intricate. This integrated model demonstrates the functional complexity of MDSCs within the tumor microenvironment and provides a novel theoretical framework for studies on the molecular mechanisms of breast cancer.

The MDSCs risk scoring system constructed in this study demonstrates clear clinical translational potential and research-promoting value. At the clinical decision-making level, this system provides clinicians with a quantitative basis for formulating individualized treatment strategies: on one hand, prognostic risk stratification assessment based on the scoring model can accurately identify high-risk patients, thereby guiding the optimization of adjuvant treatment intensity after surgery. On the other hand, the risk score shows a significant negative correlation with responsiveness to multiple chemotherapeutic and targeted drugs, which not only provides a key biological basis for developing individualized clinical treatment regimens but also assists in further screening potential beneficiaries of drugs such as camptothecin, cisplatin, dasatinib, etoposide, gemcitabine, methotrexate, paclitaxel, rapamycin, sunitinib, and vinblastine. Notably, the higher responsiveness of the low-risk cohort to chemotherapy/targeted therapy is highly consistent with the classical mechanism by which MDSCs drive chemoresistance by producing reactive oxygen species (ROS) and suppressing immune effector cells such as T cells [49]. Based on this, high-risk populations are expected to be potential priority beneficiaries of ROS scavengers combined with T-cell therapy.

In terms of immunotherapy, this study further found that low MDSCs scores are significantly associated with the immune-activated tumor microenvironment (hot tumor) phenotype—characterized by an increased CD8⁺ T cell/Treg cell ratio and a decreased TIDE score (indicating reduced risk of immune evasion). This feature highlights its potential as a predictive indicator for the efficacy of immune checkpoint inhibitors (ICIs) (AUC=0.79, indicating strong predictive ability), fully reflecting the clinical translational value of this scoring system. Notably, although the increased indicators of T-cell dysfunction in the low-risk cohort initially seem contradictory to the "hot tumor" phenotype, they may reflect the transitional stage of effector T cells from activation to exhaustion. Recent studies on the clonal proliferation of exhausted T cells after PD-1 blockade therapy have provided strong support for this hypothesis [50]. The results of this study not only validate this theory but also provide new insights into understanding the clonal dynamics of exhausted T cells after ICI treatment.

Moreover, From the perspective of dynamic clinical application, the monitoring value of this scoring system also deserves attention: for patients with poor prognosis and high risk of chemotherapy resistance, we recommend prioritizing MDSCs-targeted intervention strategies; conversely, patients with low risk who are chemotherapy-sensitive and exhibit an active immune microenvironment may derive greater clinical benefit from standardized chemotherapy-immunotherapy combination regimens. These results offer critical biological validation for the entire-course management framework of "stratification before treatment - monitoring during treatment - evaluation after treatment" in precision oncology. Furthermore, at the research-promoting level, this scoring system provides a new perspective for the functional mechanism analysis of MDSCs in breast cancer. Through correlation analysis between risk scores and key signaling pathways as well as immunosuppressive molecules, it can help explore potential targets of MDSCs-mediated treatment resistance, providing a reliable theoretical basis and validation tool for the subsequent development of MDSCs-targeted drugs and biomarker screening.

This study still has certain limitations. First, the significant heterogeneity among MDSCs subgroups means that despite the identification of a pan-MDSCs characteristic gene set, the functional associations with specific subgroups require further elucidation. Future research should delve into the specific regulatory networks of each subgroup. Second, the existing analytical framework primarily depends on retrospective analyses of multi-center public datasets, while cross-validation guarantees model robustness, prospective multi-center clinical trials and interventional studies remain essential to validate the clinical utility of the scoring system.

In conclusion, this study represents a significant theoretical breakthrough in breast cancer immune microenvironment research—innovatively identifying 209 breast cancer-specific MDSCs characteristic genes based on single-cell transcriptome data, providing invaluable experimental tools for subsequent related studies. The constructed MDSCs risk scoring system demonstrates important clinical translational value, achieving a tri-dimensional integration of prognostic prediction, chemotherapy sensitivity stratification, and immune response evaluation, offering a novel molecular tool and theoretical foundation for formulating personalized treatment strategies for breast cancer.

## Data statement

Access requests for the dataset of this study will be evaluated based on scientific merit and institutional data management policies. Requests should be submitted to the corresponding author, accompanied by a detailed description of the proposed research objectives and scientific rationale. Approval requires scientific review, adherence to institutional data access guidelines, and the signing of a data sharing agreement if necessary.

## Funding sources

Funding for this study was provided by the Guangdong Medical Research Foundation Program (Grant Number: No. A2023169), Doctoral Scientific Research Start-up Fund from Guangdong Medical University (2025) (Grant Number: No. 4SG25159G), Guangdong Basic and Applied Basic Research Fund Project - Natural Science Fund Project (Grant Number: No. 2022A1515012190), Guangdong Natural Science Fund Project (Grant Number: No. 2023A1515010448 to BBL) and Dongguan Social Development Science and Technology Program (Grant Number: No. 20231800936042 to BBL).

## Ethics statement

- Approval of the research protocol by an Institutional Reviewer Board: N/A

- Informed Consent: N/A

- Registry and the Registration No of the study/trial: N/A

- Animal Studies: N/A

## CRediT authorship contribution statement

**Jinbao Yin:** Writing – original draft, Methodology, Funding acquisition, Data curation, Conceptualization. **Binbin Li:** Visualization, Funding acquisition, Data curation. **Hui Xiong:** Visualization, Validation. **Jiepeng Gan:** Visualization, Validation. **Lan Liang:** Writing – review & editing, Supervision, Investigation.

## Declaration of competing interest

The authors confirm that there are no potential conflicts of interest related to this work. No financial or personal relationships with other people or organizations that could inappropriately influence this study have been disclosed.

## Appendix Supplementary materials

Image, application 1

Image, image 2

Image, image 3

Image, image 4

Image, image 5

Image, application 6

Image, application 7

Image, application 8

Image, application 9

Image, application 10

Image, application 11
