# Supplementary material for: Development of a single-cell derived MDSCs signature score for prognostic risk stratification and therapeutic decision guidance in breast cancer
Source: Transl Oncol. 2025 Nov 17;63:102605. doi: 10.1016/j.tranon.2025.102605 (PMC12664814; doi:10.1016/j.tranon.2025.102605)
Supplement: Supplementary file 1 [file mmc1.docx]

**Figure S1. Identification and validation of breast cancer MDSCs and their signature genes.**

1. Comparison of AUC scores calculated by the AUCell algorithm based on the literature-reported MDSC gene set between MDSC and non-MDSC cells in 21,651 myeloid cells from the integrated cohort.
2. Comparison of ssGSEA scores of the literature-reported MDSC gene set between MDSC and non-MDSC cells within the same myeloid cell population.
3. Analysis of activity level differences of classical marker genes (CD14, CD33, ITGAM, S100A8, S100A9) between MDSC and non-MDSC cells, assessed by the viper algorithm.
4. Spearman correlation analysis between the literature-reported MDSC gene set and the study-derived BRCA-MDSC gene set across bulk transcriptome datasets including TCGA-BRCA, METABRIC, and SCANB.
5. UMAP spatial distribution characteristics of AUCell activity scores of the BRCA-MDSC gene set in myeloid cells from the GSE210963 cohort.
6. BRCA-MDSC gene set AUCell activity scores in myeloid cells before and after ibrutinib treatment in the GSE210963 cohort.
7. GSEA enrichment analysis of the BRCA-MDSC gene set before and after entinostat treatment in the GSE166321 cohort (bulk data), showing normalized enrichment scores (NES) and FDR values.
8. UMAP spatial distribution characteristics of BRCA-MDSC gene set AUCell activity scores in myeloid cells from the GSE166321 cohort (scRNA data).

I. BRCA-MDSC gene set AUCell activity scores in myeloid cells before and after entinostat treatment in the GSE166321 cohort (scRNA data).

**Figure S2. Multi-cohort prognostic validation of the MDSC risk score and nomogram model performance evaluation.**

1. D. Survival prognostic validation of the MDSC risk score across multiple independent cohorts: Comparison of Kaplan-Meier survival curves between high- and low-risk groups in A (METABRIC cohort, overall survival [OS]), B (SCANB cohort, OS), C (GEO-RFS cohort, recurrence-free survival [RFS]), and D (GEO-DMFS cohort, distant metastasis-free survival [DMFS]) (log-rank test).

E-F. Analysis of independent prognostic value of the MDSC risk score in the METABRIC cohort: Univariate (E) and multivariate (F) Cox regression evaluating the prognostic correlation of the risk score and clinicopathological features (age, tumor stage, etc.) with OS (showing hazard ratio [HR] values and 95% confidence intervals [CIs]).

1. H. Analysis of independent prognostic value of the MDSC risk score in the SCANB cohort: Univariate (G) and multivariate (H) Cox regression evaluating the prognostic correlation of the risk score and clinicopathological features with OS (showing HR values and 95% CIs).
2. Calibration curve analysis of the nomogram model in the TCGA-BRCA cohort: Validating the consistency between predicted and observed probabilities of 1-, 3-, and 5-year OS.
3. Time-dependent ROC analysis of the nomogram model in the METABRIC cohort: Assessing the predictive efficacy for 1-, 3-, and 5-year OS (area under the curve [AUC]).

K. Time-dependent ROC analysis of the nomogram model in the SCANB cohort: Assessing the predictive efficacy for 1-, 3-, and 5-year OS (AUC).

**Figure S3. Functional enrichment analysis of MDSC risk score-related genes in the TCGA-BRCA cohort.**

1. Gene Ontology Biological Process (GO: BP) enrichment analysis of 292 differentially expressed genes associated with the MDSC risk score, identified in the TCGA-BRCA cohort (FDR < 0.05).

B. KEGG pathway enrichment analysis of the same 292 differentially expressed genes (FDR < 0.05).

**Figure S4. Cell-cell communication feature analysis associated with the MDSC risk score in the GSE176078 cohort (based on the CellChat algorithm)**.

1. B. Differences in intercellular subpopulation communication network features between the high MDSC risk group and low risk group: A shows comparison of the total number of communication events (red: enhanced communication events in the high-risk group; blue: reduced communication events; line thickness reflects differences in event counts). B displays differences in average interaction strength (red: increased interaction strength in the high-risk group; blue: decreased strength).

C-D. Interactive analysis of differentially activated signaling pathways between MDSC subpopulations and other cell subtypes in the high- vs. low-risk groups: C presents significantly upregulated signaling pathways (specifically enhanced in the high-risk group); D shows significantly downregulated signaling pathways (specifically attenuated in the high-risk group).
