# Supplementary figures and images for: Development of a single-cell derived MDSCs signature score for prognostic risk stratification and therapeutic decision guidance in breast cancer
Source: Transl Oncol. 2025 Nov 17;63:102605. doi: 10.1016/j.tranon.2025.102605 (PMC12664814; doi:10.1016/j.tranon.2025.102605)

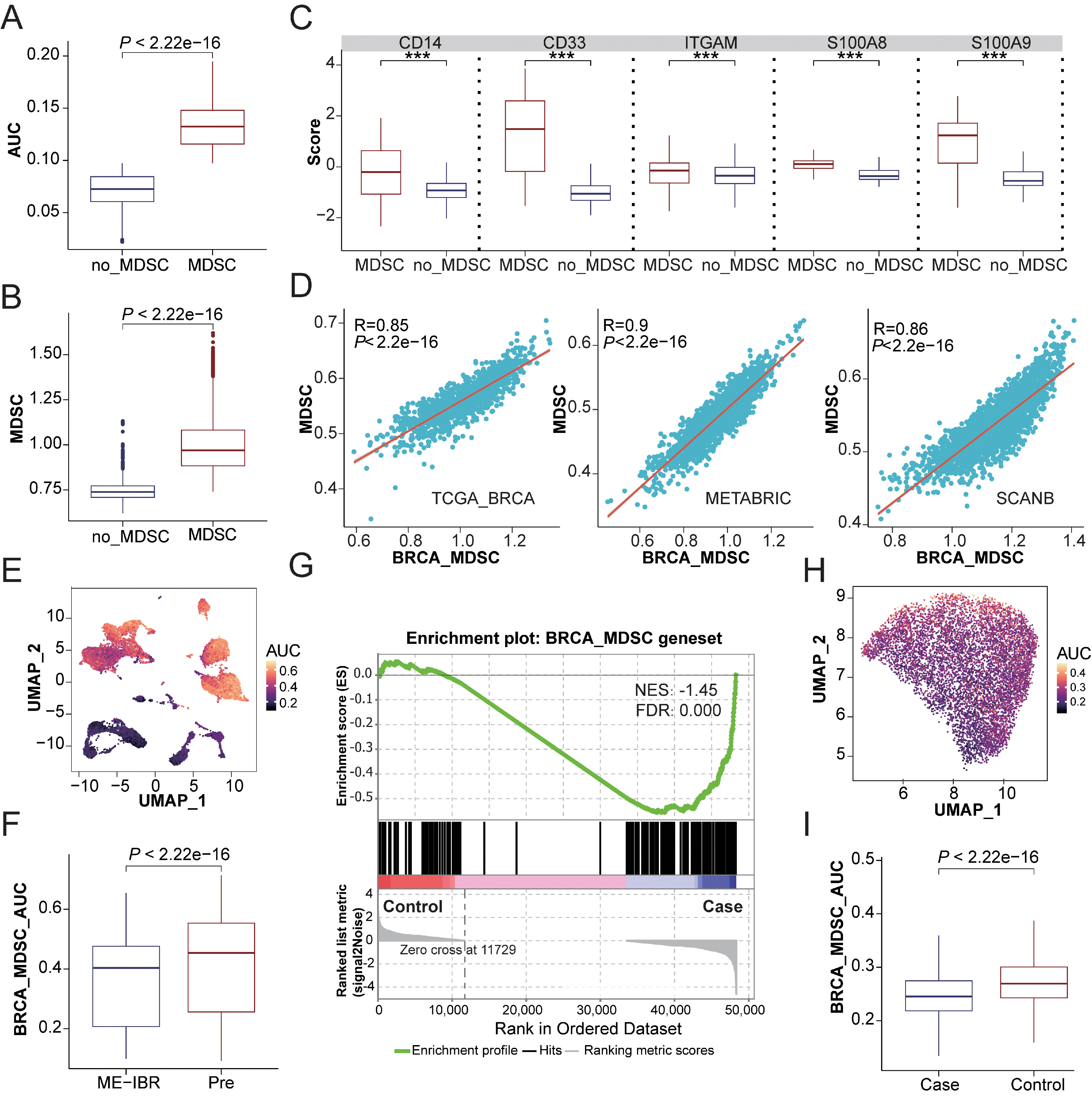

Supplement: Supplementary file 2 [file mmc2.jpg]

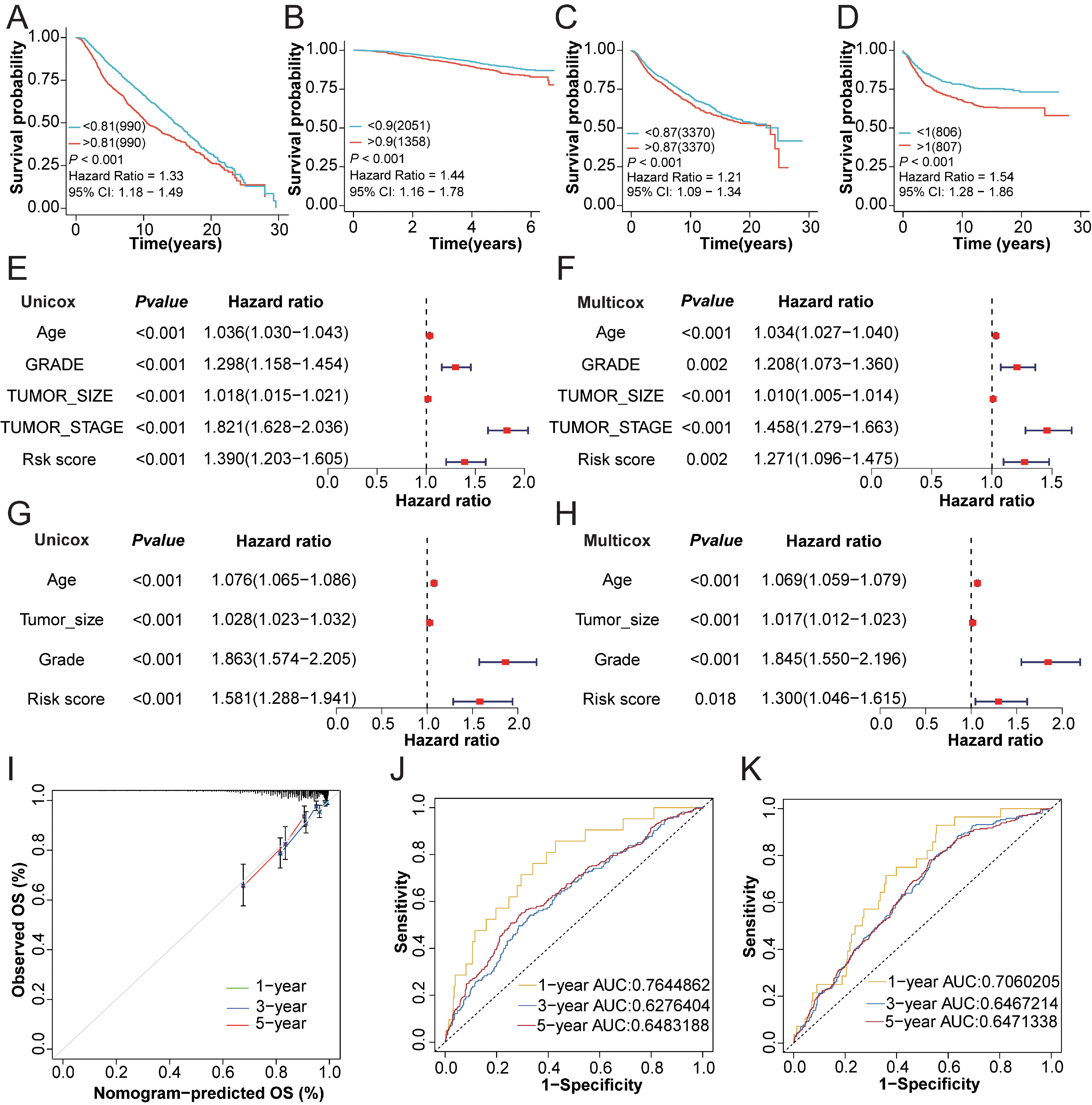

Supplement: Supplementary file 3 [file mmc3.jpg]

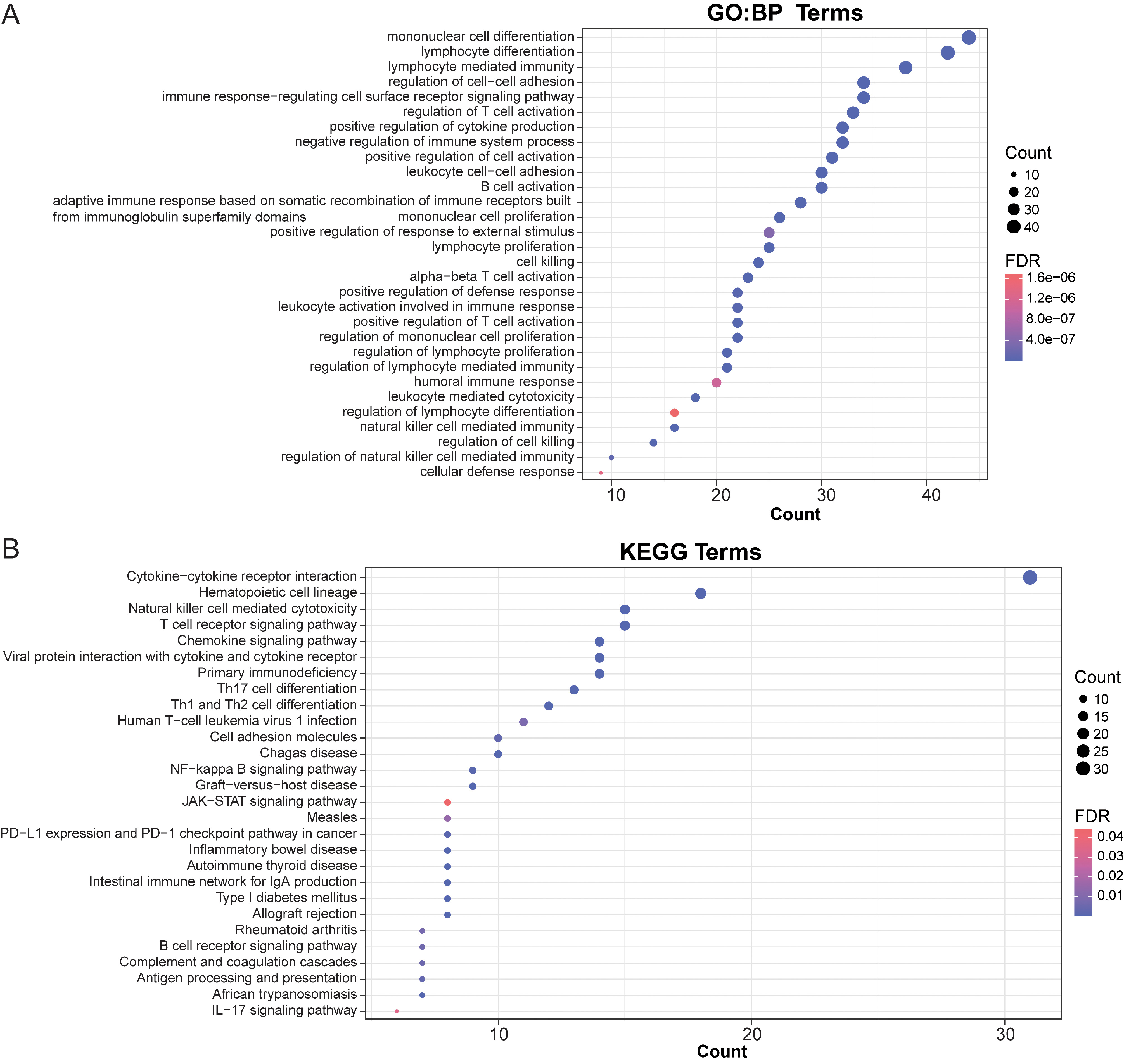

Supplement: Supplementary file 4 [file mmc4.jpg]

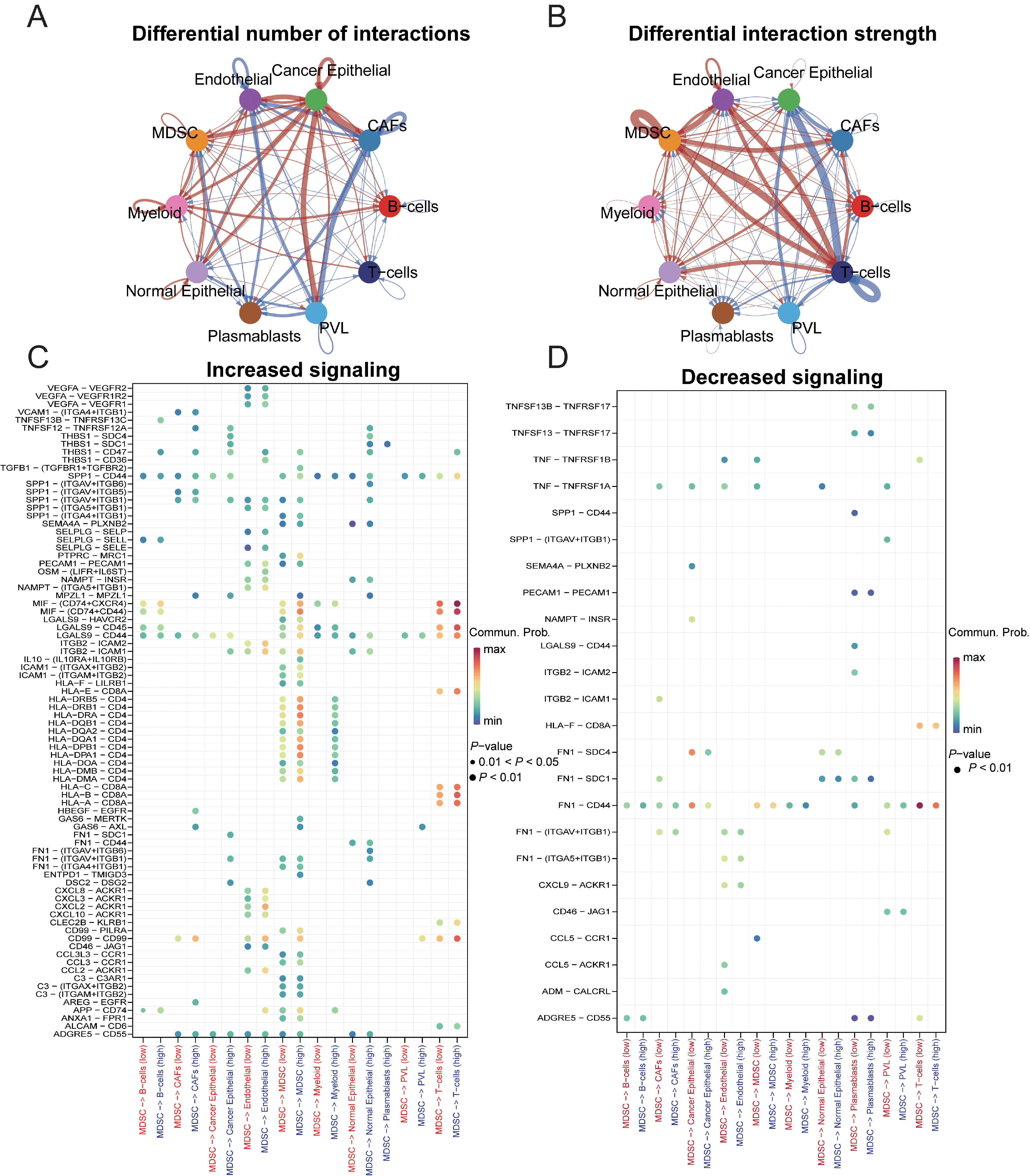

Supplement: Supplementary file 5 [file mmc5.jpg]
