# Supplementary material for: Development of a single-cell derived MDSCs signature score for prognostic risk stratification and therapeutic decision guidance in breast cancer
Source: Transl Oncol. 2025 Nov 17;63:102605. doi: 10.1016/j.tranon.2025.102605 (PMC12664814; doi:10.1016/j.tranon.2025.102605)
Supplement: Supplementary file 6 [file mmc6.doc]

**Supplementary Table1.** Detailed Information of Different Breast Cancer Datasets Involved in This Study.

**Supplementary Table2.** Characterization of a Selected Set of 312 MDSC Signature Genes Compiled from Various Sources.

**Supplementary Table3.** Profile of the BRCA-MDSCs Gene Set Defined Based on Breast Cancer Single-Cell Data in This Study.

**Supplementary Table4.** Inventory of 292 Genes Identified by Differential Expression Analysis of High- and Low-Risk Score Subgroups of MDSCs in the TCGA-BRCA Cohort.

**Supplementary Table5.** Details of Hot Tumor Phenotype and Immune Checkpoint Gene Sets Involved in Immunophenotyping Study
